# Feasibility of Silicon Quantum Dots as a Biomarker for the Bioimaging of Tear Film

**DOI:** 10.3390/nano12121965

**Published:** 2022-06-08

**Authors:** Sidra Sarwat, Fiona Jane Stapleton, Mark Duncan Perry Willcox, Peter B. O’Mara, Richard David Tilley, J. Justin Gooding, Maitreyee Roy

**Affiliations:** 1 School of Optometry and Vision Science, University of New South Wales (UNSW), Sydney 2052, Australia; s.sarwat@unsw.edu.au (S.S.); f.stapleton@unsw.edu.au (F.J.S.); m.willcox@unsw.edu.au (M.D.P.W.); 2 School of Chemistry, Australian Centre for NanoMedicine, ARC Centre of Excellence in Convergent Bio-Nano Science and Technology, University of New South Wales (UNSW), Sydney 2052, Australia; peter.omara94@gmail.com (P.B.O.); r.tilley@unsw.edu.au (R.D.T.); justin.gooding@unsw.edu.au (J.J.G.)

**Keywords:** dry eye disease, tear film, quantum dots, fluorescence imaging, artificial tears

## Abstract

This study investigated the fluorescence and biocompatibility of hydrophilic silicon quantum dots (SiQDs) that are doped with scandium (Sc-SiQDs), copper (Cu-SiQDs), and zinc (Zn-SiQDs), indicating their feasibility for the bioimaging of tear film. SiQDs were investigated for fluorescence emission by the in vitro imaging of artificial tears (TheraTears^®^), using an optical imaging system. A trypan blue exclusion test and MTT assay were used to evaluate the cytotoxicity of SiQDs to cultured human corneal epithelial cells. No difference was observed between the fluorescence emission of Sc-SiQDs and Cu-SiQDs at any concentration. On average, SiQDs showed stable fluorescence, while Sc-SiQDs and Cu-SiQDs showed brighter fluorescence emissions than Zn-SiQDs. Cu-SiQDs and Sc-SiQDs showed a broader safe concentration range than Zn-SiQDs. Cu-SiQDs and Zn-SiQDs tend to aggregate more substantially in TheraTears^®^ than Sc-SiQDs. This study elucidates the feasibility of hydrophilic Sc-SiQDs in studying the tear film’s aqueous layer.

## 1. Introduction

Dry eye disease (DED) is defined as “a multifactorial disease of the ocular surface, accompanied by loss of homeostasis, ocular surface inflammation and damage, and neurosensory abnormalities” [1]. DED is one of the most common ocular conditions worldwide and a commonly reported reason for seeking eye care [2,3,4]. According to the Tear Film & Ocular Surface Society Dry Eye Workshop II (TFOS DEWS II) epidemiology report, the prevalence of DED ranges from 5 to 50% in individuals over the age of 50, and it increases with age, sex, and Asian ethnicity [5]. Biochemical and biophysical changes in the tear film are associated with DED [2].

The tear film is a complex dynamic fluid covering the anterior ocular surface, consisting of an outer lipid layer with an underlying muco-aqueous layer [4]. The dynamic behavior of tear film can be attributed to its interactive interfaces [6] and the need for frequent blinking [7]. These interfacial characteristics hinder the study of the phase dynamics of the tear film [7]. Different simulation models have been proposed for changes to the structure and function of the tear film in DED [8,9]. Interferometry [10] and optical coherence tomography [11] have been used to study the various aspects of tear film dynamics, including the spread and thickness of the lipid layer, but how these dynamic changes impact clinical signs and tear film stability is still unknown [4]. Similarly, fluorescence microscopy has been used to study tear stability and dynamics [12]. However, issues with biological autofluorescence in the visible spectrum and photobleaching can compromise long-term fluorescence imaging [13,14].

Organic dyes provide low resolution due to narrow excitation and broad emission spectra and are susceptible to photobleaching [13]. Fluorescein has an excitation maximum of 495 nm, but the cobalt blue light sources that are used with slit lamp biomicroscopes emit visible light at 450 nm [15]. The minimum threshold for the fluorescence of the tear film is 515 nm. Therefore, diagnostic dyes with an excitation wavelength of ~500 nm should be used to obtain maximum fluorescence from the tear film [15]. Using a relatively large volume of fluorescein (10–20 μL), applied to ~4 μL of the non-stimulated tear film, may disrupt the structure and interfacial interactions of the tear film [7]. Therefore, the knowledge available regarding tear film’s interfacial dynamics, including spread, replenishment, and turnover, remains inadequate [4].

To overcome the drawbacks of imaging techniques and organic dyes, quantum dots (QDs) with an excitation range of 500 nm [16] can provide maximum fluorescence for the bioimaging of tear film [7]. QDs are surface-modified nano-sized particles (2–20 nm) that can emit bright and stable fluorescence with reduced photobleaching and superior photostability [17]. The high photoluminescence quantum yield of QDs makes them good candidates for bioimaging [18]. Cadmium selenium/zinc sulfide QDs are the most common QDs used for bioimaging [19]. However, there are concerns about the toxicity of cadmium-containing QDs [20]. QDs have been used in ophthalmology as bioimaging agents, antibacterial agents, drug delivery systems, and electrical stimulators [21]. However, concerns have been raised about their potential short-term and long-term cytotoxicity in ocular and biological systems [21]. Commercially available indium-phosphide-gallium QDs with a zinc sulfide shell have been functionalized with either hydrophilic or hydrophobic surface molecules to study the dynamics of tear film layers [7]. Again, there are issues with the cytotoxicity of indium and gallium [22].

The potential biocompatibility and ease of surface modification of silicon make photoluminescent silicon quantum dots (SiQDs) an ideal candidate for fluorescence imaging and may eliminate any potential toxicological problems [23]. SiQDs doped with transition elements enable a wide range of emission tunability and enhanced fluorescence emission intensity [24]. SiQDs can be surface-modified to be either hydrophilic or hydrophobic [25]; their characteristics may make them suitable for bioimaging the aqueous and lipid layers of the tear film. SiQDs doped with copper have been used as near-infrared luminescent probes to detect heavy metals in biological systems [26]. In addition, studies show that silicon nanomaterials (50, 100, and 150 nm) are biocompatible with human corneal epithelial cells (HCECs) [27,28,29]. There appear to be no significant cytotoxic effects of silicon nanoparticles on endothelial cells [30]. However, there is still limited information about their possible toxicity to the ocular surface [31]. The lack of toxicological information and their evaluation in standardized testing protocols limits their future applications in ophthalmology [32]. There are limited studies on the cytotoxicity of silicon nanomaterials with a size range of <20 nm to HCECs [27,28]. HCECs constitute the outermost barrier of the ocular surface and are continuously exposed to the outer environment; therefore, they provide the first line of defense against foreign agents invading the ocular surface [33,34]. Damage to HCECs may result in corneal transparency and vision loss [35]. Thus, it is essential to evaluate the potential toxicity of QDs to HCECs before their administration in an animal model or human clinical trial [36]. This study outlines the synthesis, fluorescence characterization, in vitro imaging, and cytotoxicity of three SiQDs, for their eventual application as a biomarker for studying tear film dynamics.

## 2. Materials and Methods

### 2.1. Synthesis of SiQDs

SiQDs that were doped with two or four dopant atoms per particle (Cu, Sc, and Zn) were synthesized by adding 0.5 g of tetraoctylammonium bromide and 0.026 mmol of anhydrous salt in the form of ScCl_3_, CuCl_2,_ or ZnCl_2_ to a Schlenk tube. The Schlenk tube was then attached to a Schlenk line for the triple evacuation cycle and was purged with nitrogen for 5 min per cycle. Then, 50 mL of anhydrous toluene was added, and the mixture was stirred for 24 h. Silicon tetrachloride was added to the mixture and agitated for an hour. Five equivalents of lithium aluminum hydride (LiAlH_4,_ a reducing agent) were added, then they were left to react for 3 h. This procedure yielded hydride-capped Sc-, Cu- or Zn-SiQDs. Excess lithium aluminum hydride was quenched using ethanol, which was added dropwise (until no bubbles formed).

### 2.2. Surface Passivation of SiQDs

The surface of the hydride-capped SiQDs was passivated by adding anhydrous allylamine to produce hydrophilic surfaces [25]. A quartz tube was attached to the Schlenk line, and the solution was degassed by triple cycles of evacuation and purging with nitrogen for 5 min per cycle. Hydride-capped SiQDs were transferred via a degassed syringe from the Schlenk tube to the quartz tube. Degassed anhydrous allylamine was added to the mixture, then the line was exposed to UV light for 4 h, giving propylamine (hydrophilic)-capped SiQDs.

### 2.3. Purification of SiQDs

The purification of SiQDs is essential for in vivo bioimaging where the unreacted material or side products have toxic effects [37]. After passivation, propylamine-SiQDs were transferred to a round-bottomed flask, then the solvent was removed under reduced pressure. Then, Milli-Q water was added and the mixture was dispersed by ultrasonication for 5 min, resulting in a cloudy white solution. This mixture was filtered through a 0.45 μm filter. The resulting filtrate was concentrated to 2–3 mL under low pressure and poured into a size exclusion column containing Sephadex LH-20 beads. The fractions were collected in an automated test tube collector and checked for luminescence, using a handheld UV light (365 nm). The luminescent fractions were further concentrated under reduced pressure to yield purified hydrophilic SiQDs.

### 2.4. Characterization of SiQDs

The samples were prepared for Olympus JEOL 2010 transmission electron microscopy (Olympus Life Science, Notting Hill, VIC, Australia) by drop-casting the purified doped SiQDs, suspended in 0.5–1.0 mL of ethanol, for hydrophilic SiQDs on carbon-coated copper grids. TEM images were taken at an acceleration voltage of 200 kV. The photoluminescence of each type of QD was recorded on an RF-5301PC spectrofluorophotometer (Shimadzu, Rydalmere, NSW, Australia), using an excitation and emission slit width of 3 nm. A PerkinElmer FT-IR spectrometer (Agilent, Santa Clara, CA, USA) was used to record the absorbance of the SiQDs.

### 2.5. Development of an Optical Imaging System

**Figure 1:**
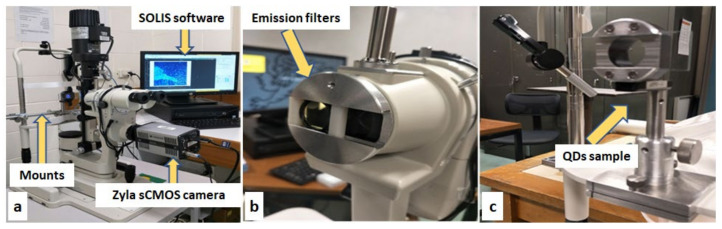
Modified slit-lamp biomicroscope for the in vitro imaging of Quantum Dots (QDs). (**a**) A Zyla sCMOS camera with SOLIS software. (**b**) The emission filter, in front of the objective lens. (**c**) QDs sample holder at the position of the chin rest.

A slit-lamp biomicroscope (Carl Zeiss, Dublin, CA, USA), a commonly used instrument to examine the human eye, was modified with a high-resolution 5.5-megapixel Zyla sCMOS camera with custom-made optical mounts, along with emission filters (460 nm, 510 nm, and 530 nm) to capture the images. The Zyla sCMOS camera offered an entire spectrograph, automatic spectral line identification, and camera control, with two- and three-dimensional data acquisition. High-quality data export options were available as two main (vertical and horizontal) binning variants, sorted into binning patterns. The SOLIS software (Andor Oxinst, Belfast, UK) was used to control the camera and capture the images. Custom-made optical mounts were built by the workshop staff at the Faculty of Science of the University of New South Wales. Optical mounts were designed to be comparable in size to the microscope slides and filters used (Figure 1). Emission filters were placed precisely in front of the objective lens, with the help of a sliding optical mount. The details of the optical imaging system are included in the Supplementary Materials.

### 2.6. In Vitro Imaging of SiQDs

Fluorescence data were measured by the in vitro imaging of Cu-SiQDs, Sc-SiQDs, and Zn-SiQDs (0.01 μg/mL to 16 μg/mL) in TheraTears^®^ (Akorn Consumer Health, Ann Arbor, MI, USA; TheraTears^®^ is a balanced electrolyte formula used as lubricating eye drops) on microscope slides. Each concentration was monitored for 20 min, and images were captured at five different time points (0, 5, 10, 15, and 20 min). Aliquots (5 μL) of the diluted SiQDs were added to microscope slides. The microscope slides were then sealed with clear nail polish to prevent the evaporation of the solution. The microscope slides were placed precisely at eye level on the chin rest of a slit lamp, with the help of the optical mounts (Figure 1). The fluorescence of SiQDs in the relative fluorescence unit (RFU) was monitored with an optical imaging system, as shown in Figure 1. Images were taken with the Zyla sCMOS camera at a frame rate of 25 per second with the increased magnification of the slit lamp (7.5×, 16×, and 35×) every 5 min. A clear microscope slide and TheraTears^®^ were used as controls. Background autofluorescence from the TheraTears^®^ was subtracted from the fluorescence value of the SiQDs before data analysis. The excitation filter that was incorporated in the slit lamp was used, while external emission filters of 460 nm, 510 nm, and 530 nm were used to compare the fluorescence emission intensities of the SiQDs.

### 2.7. Cell Culture

The HCECs (P-14) were provided by the Cell Culture Lab, School of Optometry and Vision Science, UNSW, Sydney, Australia. HCECs were cultured in a T25 flask and incubated at 37 °C in a humidified 5% CO_2_ chamber (Thermo Fisher Scientific, North Ryde, NSW, Australia) in Dulbecco’s Modified Eagle Medium (DMEM; Thermo Fisher Scientific, North Ryde, NSW, Australia) supplemented with 10% fetal bovine serum (FBS; Sigma-Aldrich, Castle Hill, NSW Australia), 0.5% dimethyl sulfoxide, 1.05 mM calcium chloride, 2 ng/mL epidermal growth factor (human, recombinant) and 1% ITS 100×. The growth of HCECs was monitored for one week, with the DMEM being replaced every 48 h. When the HCECs reached >95% confluency, the cell suspension was transferred to a 96-well plate and incubated at 37 °C in 5% CO_2,_ with the DMEM being replaced every 48 h.

### 2.8. Cell Viability Assays

Following the adhesion, the HCECs were incubated with different concentrations (250, 125, 62, 32, 16, 8, 4, 2, and 1 μg/mL) of the Cu-SiQDs, Sc-SiQDs, and Zn-SiQDs in triplicate for 24 h. The wells with DMSO and DMEM were taken as the positive and negative controls, respectively. After exposure to SiQDs, the HCECs were incubated with 200 μL of trypan blue dye and incubated at 37 °C in a humidified 5% CO_2_ chamber for 5 min. The supernatant was removed and the HCECs were washed twice with PBS. The HCECs were imaged at 4×, 10×, and 40× under an inverted light microscope (Olympus Life Science, Notting Hill, VIC, Australia). The number of stained and unstained HCECs was calculated using Image J software (Version 1.53k) [38]. The percentage of viable cells was calculated by dividing the total number of viable cells by the number of cells, multiplied by 100. Cell viability of 70% was considered a cut-off value for cytotoxicity in the trypan blue exclusion test [39]. An absorbance value of 4.5 was taken as a reference point for the MTT assay [40]. During the MTT assay, 10 μL of MTT (prepared by adding 5 mg of MTT salt per mL of PBS) was added to the HCECs and three blank wells and incubated for 2–4 h at 37 °C. The color of the MTT solution was monitored until purple precipitates were noted. Then, the supernatants were discarded, and 200 μL of dimethyl sulfoxide (10% DMSO) was added to each well to solubilize the MTT. The absorbance was measured at 570 nm. The average value from the triplicate readings was measured with a spectrophotometer (BMG Labtech, The Microplate Reader Company, Mornington, VIC, Australia). The absorbance was plotted against the concentration of SiQDs.

### 2.9. Statistical Analysis

The data values were presented as a mean (standard deviation). The normality of data was assessed using the Shapiro–Wilk test. Differences between groups were examined using the Kruskal–Wallis test, with post hoc comparisons using Dunn–Bonferroni correction. Significance was determined at *p* < 0.05.

## 3. Results

### 3.1. Size and Optical Characteristics of SiQDs

**Figure 2:**
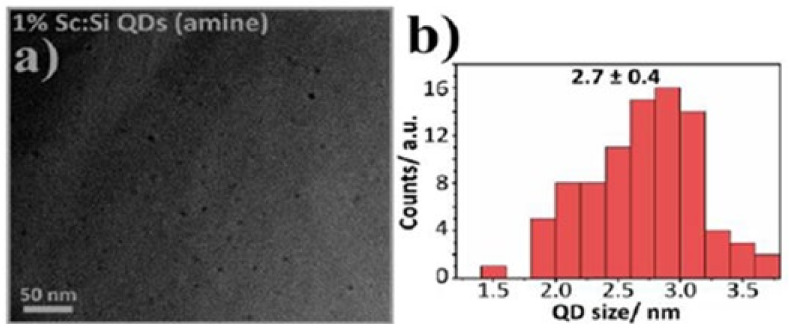
TEM image and size distribution of Sc-SiQDs. The amine-capped scandium-doped SiQDs indicate their optimal size for inducing fluorescence. Figures (**a**) and (**b**) show the TEM image (morphology) and size distribution of the SiQDs, respectively.

TEM images (Figure 2) demonstrated the spherical shape of the Sc-SiQD nanocrystals. The Sc-SiQDs, Cu-SiQDs, and Zn-SiQDs were spherical in shape and were relatively monodisperse, with an average size of 2.7 nm (0.4), 2.7 nm (0.4), and 2.6 nm (0.3), respectively.

**Figure 3:**
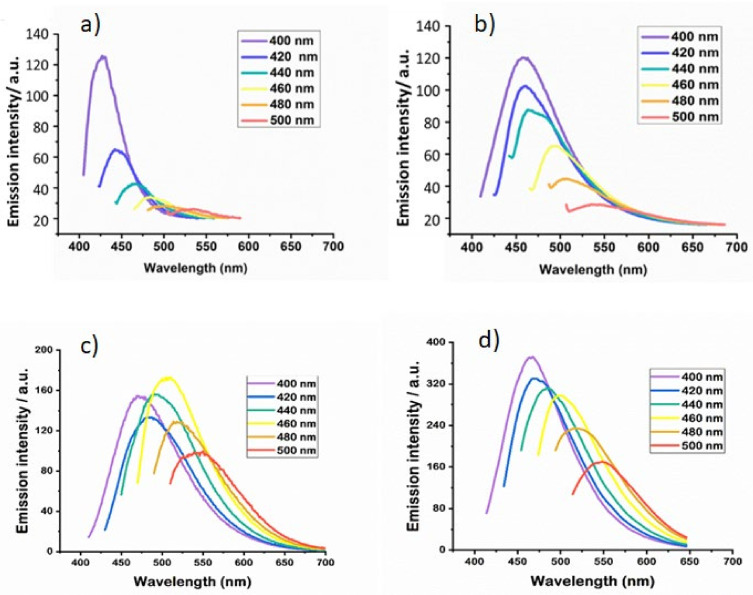
(**a**) Emission spectrum of Cu-SiQDs, using two Cu dopants per SiQD, at different exciting wavelengths (400–500 nm in 20-nm increments). (**b**) The emission spectrum of Cu-SiQDs, using four Cu dopants per SiQD at different exciting wavelengths (400–500 nm). (**c**) The emission spectrum of Sc-SiQDs at different excitation wavelengths (400–520 nm). (**d**) The emission spectrum of Zn-SiQDs at different exciting wavelengths (320–600 nm).

Figure 3a and Figure 3b show the photoluminescence emission spectra of Cu-SiQDs, doped with two and four dopant copper atoms per SiQD, at different excitation wavelengths (400–500 nm in 20 nm increments), respectively. The emission spectrum of Cu-SiQDs with four dopants shows a red-shifted (shift away from the UV-blue region) broad band with high emission intensity when using an excitation wavelength of 400 nm, in contrast to that with two Cu dopants per SiQD. A similar effect was observed on the broad emission wavelengths of the red-shifted band, with reduced emission intensity at different excitation wavelengths. The emission results show that optical properties, such as the emission wavelengths of Cu-SiQDs, may be tuned by altering the dopant concentration. Figure 3c shows the photoluminescence emission spectra of Sc-SiQDs, using a series of excitation wavelengths from 360  nm to 520 nm, in 20 nm increments. The photoluminescence emission spectra depended on the excitation wavelengths and shifted toward longer wavelengths with a narrow emission bandwidth and reduced emission intensities. The maximum photoluminescence emission peak of Sc-SiQDs was at an excitation wavelength of 460 nm, while the minimum photoluminescence emission intensity occurred at 520 nm excitation wavelength. The higher excitation wavelength has shifted the fluorescence emission peak toward a longer spectrum wavelength with reduced fluorescence intensity. The results show the optimal peak fluorescence emission at 450 nm when excited at 400 nm. The Zn-SiQDs showed a similar photoluminescence and absorption pattern to that of Cu-SiQDs and Sc-SiQDs. Figure 3d shows the photoluminescence intensities emission spectra of Zn-SiQDs, using different excitation wavelengths from 320 nm to 600 nm in 20 nm increments. As with Cu-SiQDs and Sc-SiQDs, the photoluminescence emissions are dependent on the excitation wavelengths. Hence, the photoluminescence emission spectrum shifts progressively toward the red and near-infrared with the longer excitation wavelengths.

### 3.2. In Vitro Fluorescence Imaging of TheraTears^®^ with SiQDs

**Figure 4:**
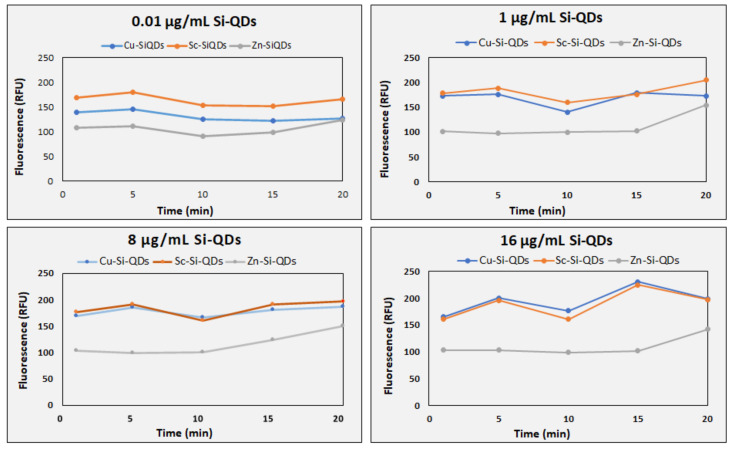
Time series of the fluorescence emission of the Cu-SiQDs, Sc-SiQDs, and Zn-SiQDs at four concentrations (0.01, 1, 8, and 16 ug/mL) at five different time points (1, 5, 10, 15, and 20 min). RFU: Relative fluorescence unit.

Figure 4 shows the fluorescence emission of Cu-SiQDs, Sc-SiQDs, and Zn-SiQDs at four different concentrations (0.01, 1, 8, and 16 ug/mL) at five time points (1, 5, 10, 15, and 20 min). Sc-SiQDs, Cu-SiQDs, and Zn-SiQDs showed a peak fluorescence emission of 270.85 RFU (23.5), 183.42 RFU (19.5), and 145.21 RFU (22.4) for five repeated measures, respectively. The Sc-SiQDs showed the highest fluorescence emission of 270.85 RFU (23.5) and remained stable at all given concentrations. Sc-SiQDs showed a minimum fluorescence of 150 RFU (0.53), still higher than the Zn-SiQDs peak value of 145.21 RFU (22.36). Although the Sc-SiQDs emitted higher fluorescence than the Cu-SiQDs, except at 0.01 μg/mL, no statistical difference was observed at any concentration (*p* = 1.98). Post hoc comparisons showed that the Sc-SiQDs and Cu-SiQDs showed significantly higher fluorescence emissions than Zn-SiQDs at 1 μg/mL and above, at the given time points (*p* < 0.001). On average, three SiQDs showed a stable fluorescence of 181.6 RFU (12.1) for 20 min (*p* = 0.15). The SiQDs showed a gradual change in fluorescence intensity up to 20 min at all given concentrations (*p* = 0.15). Zn-SiQDs reached the level of Cu-SiQDs at 20 min only when at the lowest concentration of 0.01 μg/mL. The Zn-SiQDs emitted the least bright fluorescence when compared to both Sc-SiQDs and Cu-SiQDs at all time points (*p* < 0.001).

**Figure 5:**
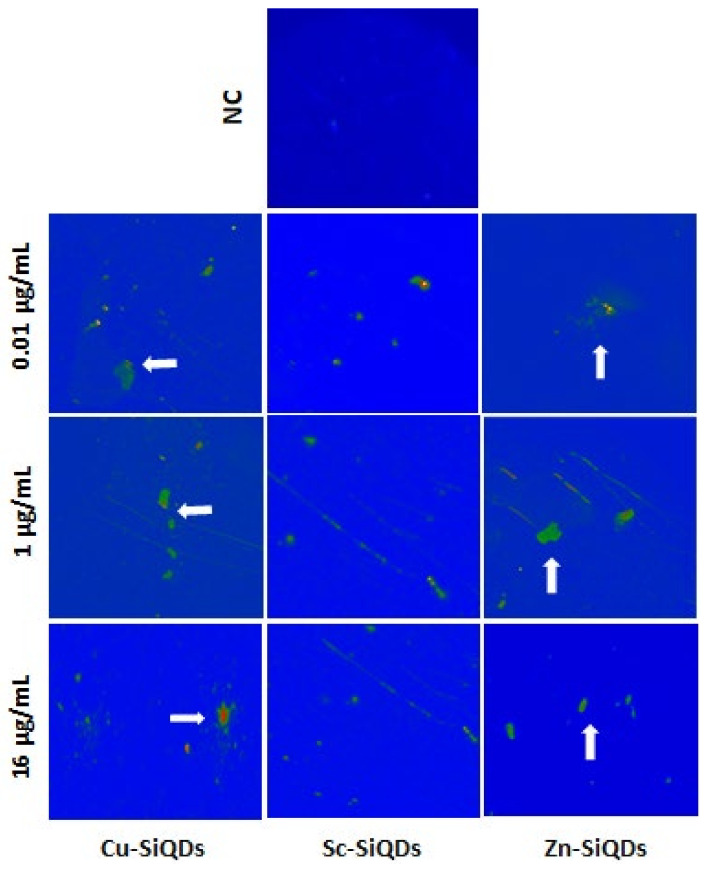
Fluorescence emissions of Cu-SiQDs, Sc-SiQDs, and Zn-SiQDs in TheraTears^®^ at different concentrations. NC = negative control (TheraTears^®^ alone); white arrowheads show the aggregations of SiQDs.

Figure 5 shows that the control (TheraTears^®^) without SiQDs gave no fluorescence signal, while the fluorescence of fluids with SiQDs was detectable at all concentrations tested. The Cu-SiQDs and Sc-SiQDs gave intense fluorescence signals, while the Zn-SiQDs tend to aggregate (white arrows on the right) at lower concentrations and showed dispersed fluorescence emission at a higher concentration of 16 μg/mL. The fluorescence intensity of Sc-SiQDs was less bright than that of Cu-SiQDs at 16 μg/mL and above (Figure 5). However, there was no statistically significant difference in the fluorescence signal intensity (*p* = 0.16). The Zn-SiQDs and Cu-SiQDs were seen to aggregate more significantly (white arrows in Figure 5). Surprisingly, the aggregation was reduced with increasing concentration, being highest at 0.01 μg/mL of Cu-SiQDs and Zn-SiQDs. The Zn-SiQDs appeared to aggregate more significantly than the Cu-SiQDs, while the Sc-SiQDs showed no aggregation behavior at any concentration (Figure 5).

### 3.3. Trypan Blue Exclusion Test

**Figure 6:**
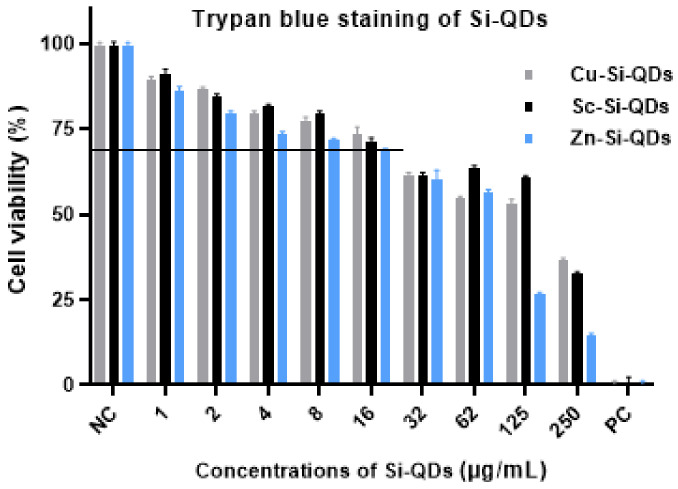
Trypan blue staining of HCECs after exposure to varying concentrations of QDs. Values are mean (SD) from three independent experiments. NC = negative control (DMEM) and PC = positive control (DMSO). The horizontal line shows the cut-off cell viability of HCECs (70%).

**Figure 7:**
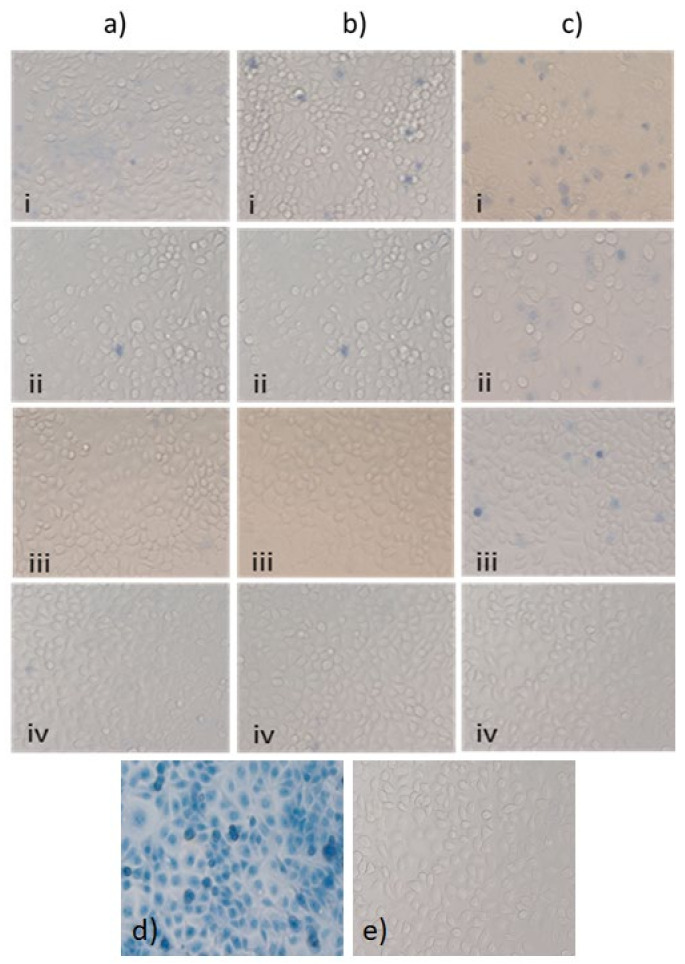
The effect of (**a**) Cu-SiQDs, (**b**) Sc-SiQDs, and (**c**) Zn-SiQDs, at (i) 250μg/mL, (ii) 32 μg/mL, (iii) 16 μg/mL, (iv) 1 μg/mL, and the (**d**) positive control and (**e**) negative control on HCECs, as assessed by the trypan blue exclusion test. (Magnification 40×).

Figure 6 and Figure 7 show the cell viability of HCECs at different concentrations of three types of QDs after 24 h of exposure, using the trypan blue exclusion test. The null hypothesis showed no difference in cell viability at all concentrations of SiQDs and the positive control. Figure 6 shows the trypan blue staining of HCECs at different concentrations of Cu-SiQDs, Sc-SiQDs, and Zn-SiQDs. HCECs have shown cell viability of <1% and >95%, in the case of the positive control and negative control respectively. The Cu-SiQDs, Sc-SiQDs, and Zn-SiQDs samples showed the lowest cell viability of 36%, 31%, and 14%, at 250 μg/mL, respectively, while showing > 95% cell viability at 1 μg/mL. Cu-SiQDs and Sc-SiQDs showed a cell viability of ≥70% at ≤16 μg/mL (*p* < 0.05); however, the Zn-SiQDs samples showed a significant decrease in cell viability at ≥16 μg/mL (*p* < 0.01). There was no statistically significant difference in cell viability among Cu-SiQDs, Sc-SiQDs, and Zn-SiQDs, at individual concentrations of 32 μg/mL and above (*p* = 1.2). The Cu-SiQDs and Sc-SiQDs showed no significant difference in cell viability at all concentrations (*p* > 0.01). However, the Zn-SiQDs samples showed significantly greater cell death than both Sc-SiQDs and Cu-SiQDs samples at ≥ 64 μg/mL (*p* < 0.05). There was a dose-dependent effect on the cell death of the HCECs, up to 125 μg/mL of Cu-SiQDs and Sc-SiQDs. There was a significant reduction in cell viability in Zn-SiQDs at 250 μL (*p* < 0.01). Zn-SiQDs showed a 50% decrease in cell viability at >62 g/mL concentration. However, Sc-SiQDs and Cu-SiQDs have shown a 50% decrease in cell viability only at 125 g/mL concentration. Overall, Cu-SiQDs and Sc-SiQDs showed no significant effect on the cell viability of HCECs at ≥16 μg/mL (*p* > 0.05), while Zn-SiQDs showed a safe concentration of ≥8 μg/mL.

### 3.4. MTT Staining

**Figure 8:**
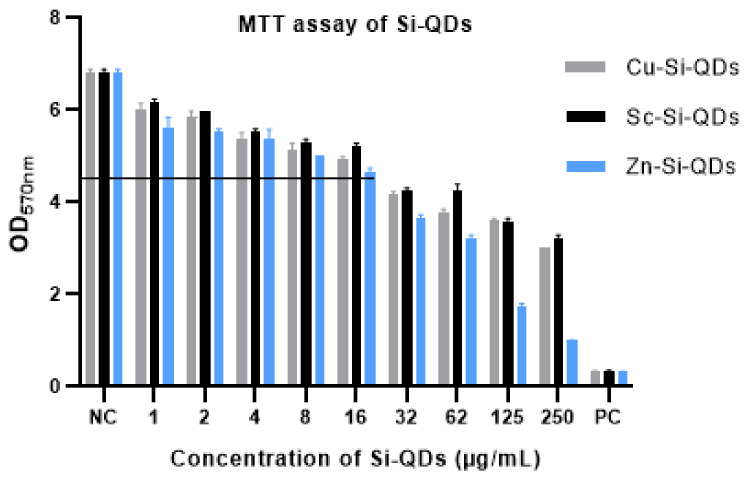
OD570 values of formazan from HCECs, after exposure to given concentrations of QDs. Values are mean (SD) from three independent experiments. NC = negative control (DMEM) and PC = positive control (DMSO). The horizontal line shows a cut-off OD 570 nm value of 4.5, indicating cell viability.

Figure 8 shows the absorbance values of formazan, where HCECs were exposed to different concentrations of Cu-SiQDs, Sc-SiQDs, and Zn-SiQDs. The positive control resulted in the lowest OD value (0.33), indicating a <5% cell viability of HCECs. The negative control showed the highest OD value (6.86), compared to a positive control (*p* < 0.05), showing the >95% cell viability of HCECs. The MTT assay produced similar results to the trypan blue exclusion test, except that Zn-SiQDs showed no effect on cell viability at ≥ 16 μg/mL, equivalent to that of the Cu-SiQDs and Sc-SiQDs.

The Cu-SiQDs, Sc-SiQDs, and Zn-SiQDs showed an average OD_570_ value of 6, indicating a >95% cell viability at 1 μg/mL and <5% cell viability at 250 μg/mL, compared to the positive control. The Cu-SiQDs, Sc-SiQDs, and Zn-SiQDs showed no difference in the reduction of cell viability in HCECs at individual concentrations of ≥32 μg/mL, compared to the positive control (*p* = 0.15). Like the trypan blue exclusion test, the Sc-SiQDs and Cu-SiQDs showed similar cell viability at all concentrations (*p* > 0.05). However, the Zn-SiQDs showed a more significant reduction in cell viability at ≥32 μg/mL than Sc-SiQDs and Cu-SiQDs. Overall, the Cu-SiQDs, Sc-SiQDs, and Zn-SiQDs showed no significant effect on the cell viability of HCECs at ≥16 μg/mL.

## 4. Discussion

Dry eye disease is one of the most prevalent ocular conditions worldwide, affecting the sufferer’s quality of life and resulting in significant economic costs [5]. The tear film plays a significant role in DED [2,4]; however, its fundamental dynamics, including spreading, replenishment, and turnover, are not fully understood [4]. QDs may be used as a biological marker to study tear film dynamics [7]. QDs offer an alternative to organic dyes because of their unique optical properties [13]. These have already been used for in vitro and in vivo bioimaging [18,40,41]. QDs possess size-tunable fluorescence, high quantum yield, and enhanced photostability [36]. However, a significant concern that may impact their use in a biological system is their potential toxicity [20]. In this study, we explored the fluorescence emission and biocompatibility of SiQDs for their eventual application as a biomarker for the study of tear film dynamics.

SiQDs were synthesized in the solution phase [25] to produce hydrophilic Cu-SiQDs, Sc-SiQDs, and Zn-SiQDs. A potent reducing agent (LiAlH_4_) yielded small-sized (2–4 nm) nanocrystals, which are desirable for the quantum yield effect and bright fluorescence emission [37]. The size and morphology of the SiQDs matches those reported in a previous study [42]. This size range (<10 nm) is desirable for efficient biodistribution and clearance from biological systems [43]. This size has also enabled a broad absorption range of 400–590 nm and a narrow emission range (400–460 nm) in SiQDs, which are suitable for visible fluorescence [42]. The visible excitation range of SiQDs has allowed the use of a visible light source in the form of a slit lamp biomicroscope for the in vitro imaging of TheraTears^®^, as previously used for QD bioimaging [7]. Additionally, the surface of SiQDs was modified with the amine group to make them hydrophilic, making SiQDs suitable for specific labeling of the tear film’s aqueous layer [7].

The fluorescence of SiQDs was detectable at all the concentrations tested, and the results were consistent with those in previous studies [25,42,44]. The Cu-SiQDs and Sc-SiQDs showed brighter fluorescence as a function of time than did Zn-SiQDs. The SiQDs showed considerably stable fluorescence for 20 min, indicating their use for more extended periods of tear film bioimaging. This result suggests the potential clinical applicability of SiQDs in the diagnosis of DED and the classification of its subtypes [7]. Fluorescence emission can be enhanced by increasing the number of dopants per QD [24]. A dopant amount of more than two atoms per SiQD increases the fluorescence emission intensities due to the enhanced quantum-yield effect [42]. Density function theory calculations show that the redshift relates to the interactions between dopant states and energy levels in the nanocrystals. This explains why there is a greater red shift with the inclusion of more dopant atoms [42]. The current study has also indicated greater fluorescence emission intensities from four-doped copper atoms than two-doped copper atoms in SiQDs, which is consistent with another study [24].

Zn-SiQDs, and Cu-SiQDs, to a lesser extent, aggregate at specific concentrations; this has been reported previously as an issue with QDs [45]. This aggregation may be due to their functional groups (propylamine), which might have attracted the components in TheraTears^®^. The aggregation of QDs can be addressed by dispersion in a suitable surfactant [46], as it will provide stability and uniform dispersion [45]. However, surfactants may disrupt the tear film during in vivo bioimaging [47]. Encapsulation and surface modification are the most common methods used to increase the stability and inertness of QDs [48]. Therefore, the modification of SiQDs with functional groups, such as PEG, may enhance their dispersion in the tear film lipid layer to avoid aggregation during bioimaging [49].

The current study suggested that SiQDs did not induce significant cytotoxicity in cultured HCECs at concentrations of up to 16 μg/mL. SiQDs showed a concentration-dependent decrease in the cell viability of HCECs above 16 μg/mL. A concentration-dependent decrease in cell viability was also observed when CdSe/ZnS QDs were applied to *Xenopus* embryos [41]. Some studies reported no significant toxic effects by SiQDs [27,28], while a few have reported higher toxicity with smaller QDs (4.6 and 6 nm) [50], and others have no toxicity, even with large-sized QDs (150 nm) [51]. A release of cadmium ions within cells is a prominent cause of cytotoxicity by cadmium tellurium QDs without a cadmium sulfide shell [20]. It is conceivable that the ZnS shell of the SiQDs reduced the toxic effect. Cadmium tellurium QDs (2.2 nm) showed no significant impact on the cell viability of L929 fibroblast cells at <7 μg/mL [52].

In contrast to the present study, where 50% cell death was observed at 62 μg/mL in HCECs, it was reported previously that the in vitro cytotoxicity of CdSe/ZnS QDs caused 50% cell death of corneal stromal fibroblasts at 0.5–5 μg/mL [50]. Similarly, large-sized silicon oxide nanoparticles (50, 100, and 150 nm) showed no acute toxic effects at a concentration of 100 μg/mL on cultured HCECs, following 48 h of exposure [27,28]. Conversely, small-sized gold nanoparticles (20 nm) did not affect retinal endothelial cells in mice, even at a concentration of 1 g/kg [53]. These studies suggest the effects of size, concentration, cell type, and exposure time on the cytotoxicity of SiQDs [54], which explains the lack of consensus in the literature. Further studies should assess the cytotoxic effects of SiQDs on immortalized and primary ocular surface cells, such as conjunctival cells and meibomian gland cells. It is recommended that further studies should investigate the residence time and elimination of SiQDs in ocular surface cells.

## 5. Conclusions

In this paper, Sc-SiQDs, Cu-SiQDs, and Zn-SiQDs have been successfully synthesized, and their optimal fluorescence and cytotoxicity investigated. The optimal size (2–4 nm), fluorescence emission, and biocompatibility achieved demonstrate the possible use of SiQDs for bioimaging of tear film imaging. The Cu-SiQDs and Sc-SiQDs showed brighter fluorescence and a lesser reduction in cell viability than the Zn-SiQDs. The Cu-SiQDs and Zn-SiQDs showed aggregation behavior in TheraTears^®^. Hence, Sc-SiQDs appeared to be a better option for the future in vivo bioimaging of tear film. Still, the real challenge is now to deliver the hydrophobic SiQDs to the lipid layer of the tear film without using a toxic organic solvent such as hexane, which needs further research. Preclinical cytotoxicity tests are also recommended in an animal model.

## Supplementary Materials

The following supporting information can be downloaded at: https://www.mdpi.com/article/10.3390/nano12121965/s1, Figure S1: Components of an optical imaging system: (a) The optical imaging system was composed of a slit lamp biomicroscope, sCMOS Zyla Camera, emission filters, optical mounts and SOLIS software. (b) Emission filters placed exactly in front of the objective lens with the help of a sliding optical mount and QDs sample; Table S1: List of instruments and materials used during in vitro imaging of Si-QDs. References [55,56,57] are cited in the Supplementary Materials.
