# Supplementary material for: Feasibility of Silicon Quantum Dots as a Biomarker for the Bioimaging of Tear Film
Source: Nanomaterials (Basel). 2022 Jun 8;12(12):1965. doi: 10.3390/nano12121965 (PMC9231315; doi:10.3390/nano12121965)
Supplement: Supplementary file 1 [file nanomaterials-12-01965-s001.zip › nanomaterials-1727960-supplementary.pdf]

nanomaterials

Supplementary Materials

Feasibility of Silicon Quantum Dots as a Biomarker for the Bioimaging of Tear Film

Sidra Sarwat [1], Fiona Jane Stapleton [1], Mark Duncan Perry Willcox [1], Peter B. O'Mara [2], Richard David Tilley [2], J. Justin Gooding [2] and Maitreyee Roy [1,*]

[1] School of Optometry and Vision Science, University of New South Wales (UNSW), Sydney 2052, Australia; s.sarwat@unsw.edu.au (S.S.); f.stapleton@unsw.edu.au (F.S.); m.willcox@unsw.edu.au (M.W.)
[2] School of Chemistry, Australian Centre for NanoMedicine, ARC Centre of Excellence in Convergent Bio-Nano Science and Technology, University of New South Wales (UNSW), Sydney 2052, Australia; peter.omara94@gmail.com (P.O.); r.tilley@unsw.edu.au (R.D.T.); justin.gooding@unsw.edu.au (J.J.G.)
* Correspondence: maitreyee.roy@unsw.edu.au

S1. Development of an Optical Imaging System Using Slit-Lamp Biomicroscope

The slit lamp biomicroscope is an instrument commonly used in clinical practice [1], consisting of a high-intensity light source that can be focused on ocular structures. The slit lamp facilitates the examination of the ocular structures such as eyelids, sclera, tear film, iris, lens, and cornea, and provides a stereoscopic view of these structures [2]. The slit lamp can be utilized to examine the vitreous, choroid, and retina using a hand-held high magnification lens [3]. A slit lamp biomicroscope is equipped with two light sources, variable flash intensity, changeable illuminations, and magnification. A slit lamp biomicroscope (Carl Zeiss, Dublin, CA, USA) was modified with a high-resolution 5.5-megapixel Zyla sCMOS camera (Andor Technology Ltd, Belfast, UK), an optical mount, and emission filters (460 nm, 510 nm, and 530 nm) to capture the image the fluorescence emission from Si-QDs.

The Zyla sCMOS camera can split the detection path thus allowing 100% of the reflected light to enter the camera. This enables visualization and photography of subtle details of the ocular structures.

The camera has the following specifications:

- Full spectrograph and camera control
- 2D and 3D data acquisition
- High quality data export options are SIF, TIFF, Text, Data and FITS.
- Automatic spectral line identification
- Two main (vertical and horizontal) binning variants to give binning patterns

The Zyla sCMOS camera has high quantum efficiency and spectral response with background light correction. SOLIS software was used to control the camera and capture the images. SOLIS (Andor Technology Ltd, Belfast, UK) is Andor's camera control and analysis software specifically designed for imaging and offers rich functionality for data acquisition and processing. In addition, custom made optical mounts were built by the workshop staff at the Faculty of Science, UNSW, Sydney, Australia. Optical mounts were designed to be comparable in size to the microscope slides used (Figure S1a,b). Emission filters were placed exactly in front of the objective lens with the help of a sliding optical mount (Figure S1b).

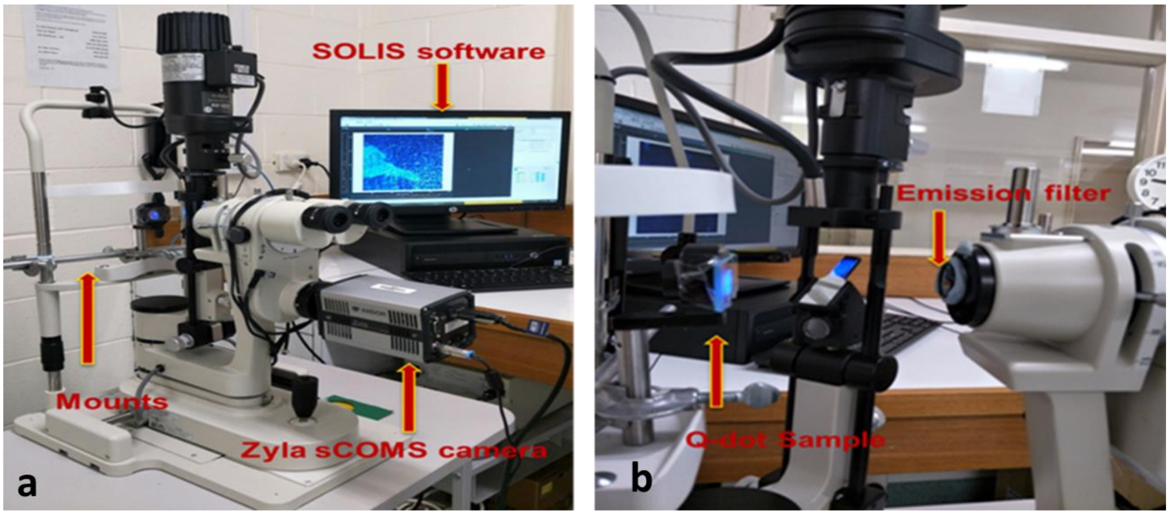

Figure S1. Components of an optical imaging system: (**a**) The optical imaging system was composed of a slit lamp biomicroscope, sCMOS Zyla Camera, emission filters, optical mounts and SOLIS software. (**b**) Emission filters placed exactly in front of the objective lens with the help of a sliding optical mount and QDs sample.

Fluorescence of the Si-QDs was explored by imaging 15 different concentrations from 0.01 µg/mL to 250 µg/mL (serial dilution method) of the Cu-Si-QDs, Sc-Si-QDs and Zn-Si-QDs in TheraTears® (a balance electrolyte formula used as lubricating eye drops) on microscope slides. Each concentration was monitored for 20 min, and images were captured at five different time intervals (1 min, 5 min, 10 min, 15 min & 20 min). Aliquots (10 µL) of the diluted Si-QDs were added to microscope slides. The microscope slides were then sealed with clear nail polish to prevent the evaporation of the solution. The microscope slides were placed exactly at the level of the eyes on the chin rest of a slit lamp with the help of the optical mounts Figure S1. The fluorescence of Si-QDs was monitored with an optical imaging system as shown in Figure S1. Images were taken with the Zyla sCMOS camera at a frame rate of 25 per second with increasing magnification of the slit lamp (7.5×, 16×, and 35×) every 5 min. A clear microscope slide and TheraTears® were used as controls. The background autofluorescence from the TheraTears® was subtracted from the fluorescence value of Si-QDs and then statistically analysed. The excitation filter incorporated in the slit lamp was used, while external emission filters 460 nm, 510 nm and 530 nm were used to compare the fluorescence emission intensities of the Si-QDs.

Table S1. List of instruments and materials used during in vitro imaging of Si-QDs.

Reagent or instrument	Provider
Slit lamp bio-microscope	Carl Zeiss Meditec, Inc. USA
sCOM camera	Andor Technology Ltd, Belfast, UK
SOLIS software (I)	Andor Technology Ltd, Belfast, UK
TheraTears®	Akorn, Inc., Ann Arbor, MI, USA
Microscopic slides (25 mm × 75 mm).	Livingstone Laboratories, Australia
Microscopic cover slips	Livingstone Laboratories, Australia

References

1. Al-Abdulla N, Kerrison J. Use of the slit lamp biomicroscope for examining ocular anatomy and pathology. *Topics in Emergency Medicine* **2000**, *22*, 52–57.
2. Martin, R. Cornea and anterior eye assessment with slit lamp biomicroscopy, specular microscopy, confocal microscopy, and ultrasound biomicroscopy. *Indian J. Ophthalmol.* **2018**, *66*, 195–201, https://doi.org/10.4103/ijo.ijo_649_17.
3. Lee, N.B. Biomicroscopic examination of the ocular fundus with a +150 dioptre lens. *Br. J. Ophthalmol.* **1990**, *74*, 294–296, https://doi.org/10.1136/bjo.74.5.294.
